# Climbing Mechanism Design and Fuzzy PID-Based Control for a Stay Cable De-Icing Robot

**DOI:** 10.3390/s25216765

**Published:** 2025-11-05

**Authors:** Yaoyao Pei, Shunxi Li, Zhi Chen, Henglin Xiao, Silu Huang, Changjie Li, Lei Xi

**Affiliations:** 1 School of Civil Engineering, Hubei University of Technology, Wuhan 430068, China; shunxilia@163.com (S.L.); chenzhi1988420@126.com (Z.C.); xiao-henglin@163.com (H.X.); leixi@whut.edu.cn (L.X.); 2 State Key Laboratory of Precision Blasting, Jianghan University, Wuhan 430056, China; 3 Hubei Jiaotou Intelligent Testing Co., Ltd., Wuhan 430050, China; 19571706880@163.com (S.H.); 18827344003@163.com (C.L.)

**Keywords:** stay cable, climbing mechanism, clamping force, fuzzy PID control

## Abstract

In winter, ice is prone to forming on the surface of stay cables in cable-stayed bridges, posing a threat to their structural safety. As temperatures rise, the risk of ice shedding increases, posing a potential hazard to pedestrians and vehicular traffic. At present, de-icing relies mainly on manual operations, which are associated with high safety risks and low efficiency. As a result, the application of robotic systems for stay cable de-icing has become an emerging research focus. A key challenge in robotic de-icing operations lies in the complex and variable surface conditions of ice-covered stay cables, which frequently hinder stable climbing performance. To address this issue, a climbing mechanism was designed, integrating a grooved-track drive and a spring-assisted lead screw clamping system. A fuzzy PID control strategy was implemented to achieve adaptive coordination between the clamping force and climbing speed. Simulink simulations and indoor climbing experiments were performed to verify its effectiveness. The results show that compared with traditional PID control, the fuzzy PID controller reduces the response time by approximately 50%, exhibits better adaptability in icy environments, maintains a climbing speed error within ±1.5%, and improves overall climbing performance.

## 1. Introduction

**Figure 1:**
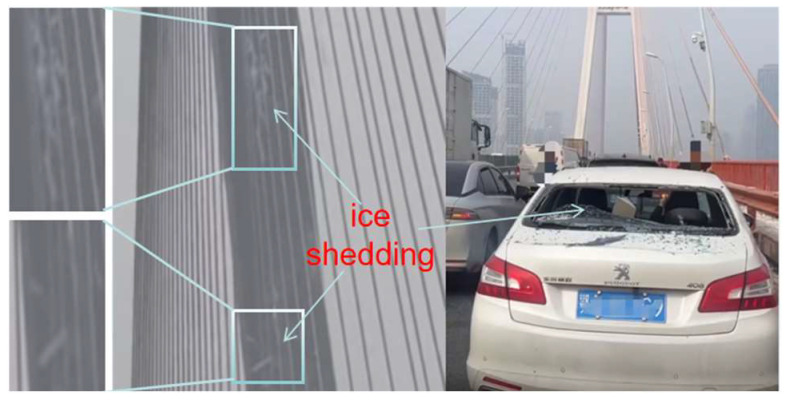
Safety risks induced by ice shedding.

Ice accretion on the surface of stay cables in cable-stayed bridges is common in winter [1,2]. Ice accretion not only increases the additional load on the stay cables and alters the aerodynamic characteristics of the bridge superstructure, but also induces irregular cable vibrations, thereby posing a risk to the structural safety of the bridge [3,4,5]. Moreover, as ambient temperatures rise, accumulated ice may detach and fall from great heights, endangering pedestrians and vehicles on the bridge deck [6,7]. In recent years, with the rapid proliferation of cable-stayed bridges, ice-shedding incidents have become more frequent, resulting in significant economic losses and serious safety hazards [8], as shown in Figure 1.

**Figure 2:**
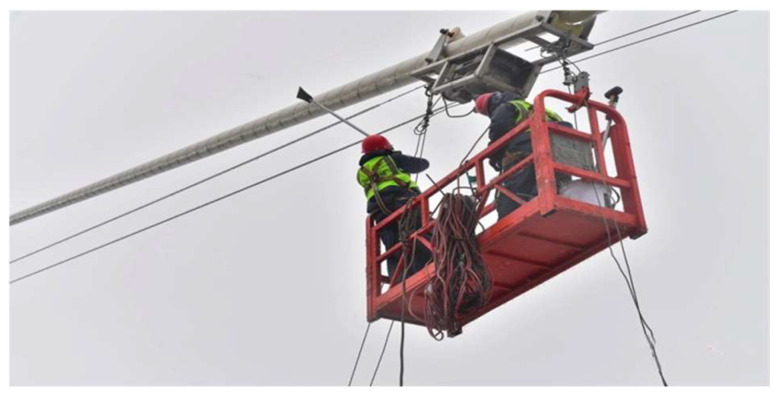
Stay cable de-icing by manual operation.

Currently, no effective strategy has been implemented. Manual de-icing is inefficient, complex, and poses high risks due to elevated operations, as shown in Figure 2. Although anti-icing coatings have been discussed in the literature [9], no practical application on stay cables has been reported; most studies remain at the laboratory stage [10]. Under these circumstances, applying robotic technology to stay cable de-icing presents an effective and innovative solution. During operation, the stay cable de-icing robot is required to climb along the surface of ice-covered cables, and its stable performance depends on both the climbing mechanism and the control system. The climbing mechanism must accommodate the reduced adhesion and fluctuating friction caused by surface icing to ensure reliable traction and continuous climbing capability. Meanwhile, the control system must provide real-time sensing and adaptive regulation to cope with severe variations in the friction coefficient and frequent load disturbances [11]. In addition, research on soft robotics has made significant progress in adaptive control under complex and nonlinear conditions. Mao et al. [12] proposed a machine-learning-enhanced, bio-inspired soft robotic system capable of achieving high-precision control under multi-field coupling conditions, which provides valuable inspiration for the design of the fuzzy PID control strategy developed in this study. However, existing research specifically targeting stay cable de-icing robots remains limited, with most relevant studies focusing on related fields such as cable inspection, snow removal, or de-icing operations on power transmission lines. Existing climbing mechanisms include the dual-module design, which is designed to achieve stable adhesion to the cable by symmetrically enclosing it [13,14]. In subsequent work, this mechanism was applied to stay cable maintenance robots, where it was integrated with inspection and repair devices to enable surface damage detection and restoration tasks [15]. A cable inspection robot, consisting of a driving unit and a passive unit connected via four rotary locks, was developed. The wheels were designed with a V-shaped groove to increase the contact area and improve frictional performance [16]. A ski-assisted mechanism was developed for snow removal and surface inspection of stay cables [17]. A gripper-based mechanism capable of stable grasping and flexible locomotion along stay cables was also proposed [18,19,20]. In terms of control systems, a fuzzy PID-based control algorithm has been shown to enable adaptive regulation of speed and clamping force [21]. A fuzzy PID control strategy that combines the nonlinear regulation capability of fuzzy inference with the fast response characteristics of traditional PID control has also been developed. The fuzzy PID controller was designed with climbing speed as the input and motor current and support angle as the outputs, enabling real-time regulation of a magnetorheological damper–spring coupled loading mechanism [22]. However, most of the aforementioned climbing mechanisms are designed for use in specific and relatively simple scenarios, making them unsuitable for operation on ice-covered stay cables. Although several studies have addressed snow-covered conditions, such as the snow removal robot developed with wheeled mechanisms, these systems are prone to slipping on low-adhesion icy surfaces and, thus, are not suitable for de-icing tasks on stay cables. Additionally, no existing studies have integrated fuzzy PID control strategies with climbing mechanisms explicitly designed for icing conditions. In contrast, de-icing operations on stay cables must contend with highly demanding scenarios—such as ice-covered surfaces and complex environmental disturbances—which impose stricter requirements on both the clamping performance of the mechanism and the adaptability of the control system. These challenges highlight the urgent need to develop climbing mechanisms and control strategies tailored to the harsh conditions of stay cable icing.

In this study, a climbing mechanism for ice-covered stay cables was developed. The drive module was equipped with grooved tracks, which enhanced friction and thereby prevented undesired rotation of the machine [23], as well as with DC-geared motors. The clamping module was constructed from a lead screw stepper motor and springs. The clamping force was automatically adjusted by controlling the extension length of the lead screw, which effectively prevented the robot from jamming during climbing. A control model of the de-icing robot was established, and system simulations were conducted using MATLAB/Simulink (Version R2018a). To enhance control precision, a fuzzy PID control strategy was integrated into the existing control module, enabling dynamic adjustment of control parameters. The effectiveness of the proposed strategy was validated through simulations and climbing experiments. In addition, this study further investigates, from an application perspective, the adaptability and potential advantages of fuzzy PID control under working conditions characterized by large cable inclination, low adhesion, and ice-induced disturbances, thereby clarifying the technical characteristics and engineering significance of the proposed method in complex stay cable de-icing scenarios. The remainder of this paper is organized as follows: Section 2 presents the design of the climbing mechanism. Section 3 analyzes the relationship between the robot’s climbing speed and clamping force. Section 4 presents the simulation of the control system and the climbing experiments. Section 5 provides the conclusions of this study. Section 6 discusses the limitations and outlines directions for future research.

## 2. Description of the Prototype

### 2.1. Concept

**Figure 3:**
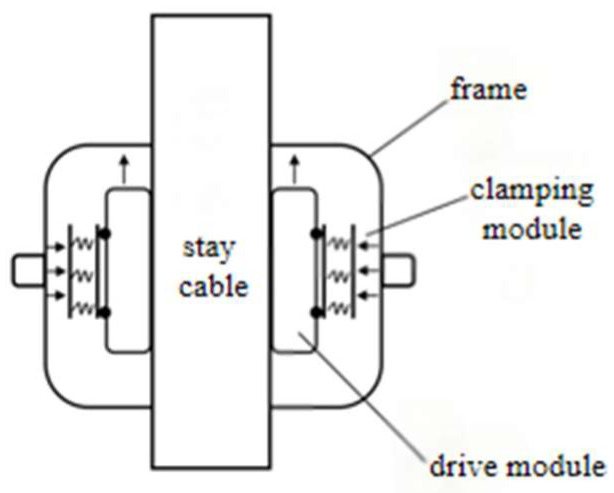
Schematic diagram of the robot design concept.

**Figure 4:**
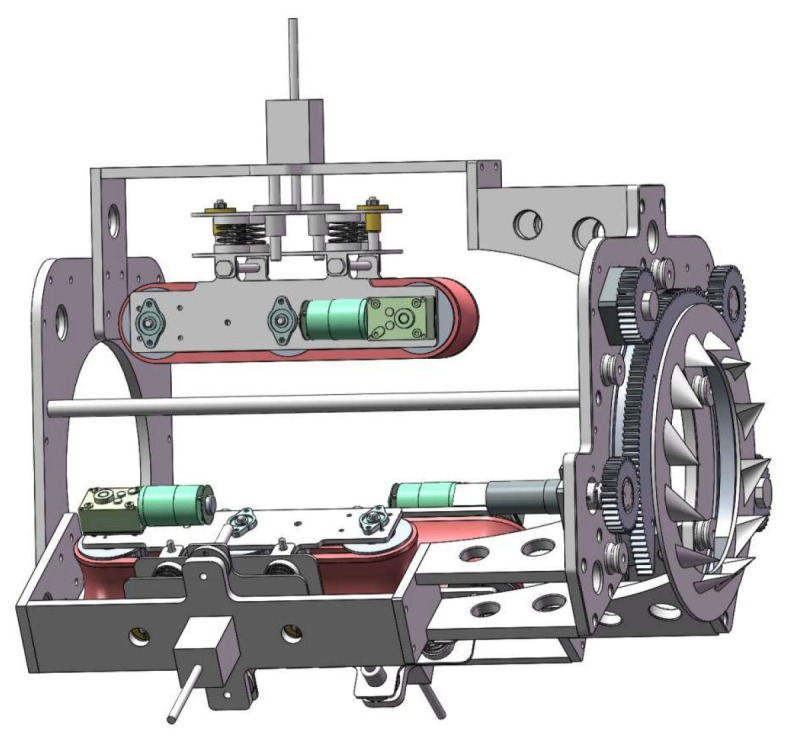
Three-dimensional model of the de-icing robot.

The design aims to develop a climbing mechanism that maintains stable movement on ice-covered cables while avoiding instability or slippage caused by insufficient or excessive friction between the robot and the stay cable. The robot comprises three identical climbing modules arranged at 120° intervals. Each module consists of a grooved track–based driving module and a clamping module. A de-icing module is installed at the front of the robot. The de-icing operation is performed by a high-speed rotating ice blade driven by a motor. The de-icing blade disk features an open-loop design, allowing the robot to be easily mounted onto the stay cable and securely clamped in position using a lead screw stepper motor, thereby enabling rapid installation. Figure 3 shows the schematic diagram of the de-icing robot’s design concept, and Figure 4 presents the 3D model of the de-icing robot.

### 2.2. Design

**Figure 5:**
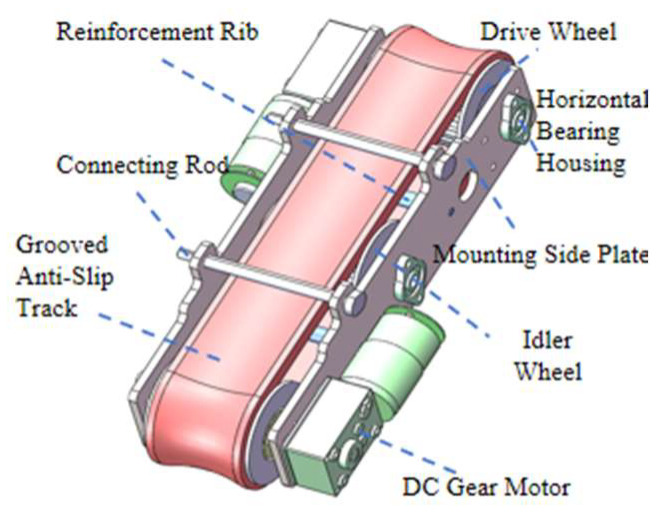
Structural diagram of the robot drive mechanism.

As shown in Figure 5, the drive mechanism consists of two driving wheels, a central idler, and a continuous track, forming a closed traction loop. The track-based design increases the contact area between the robot and the stay cable. The inner grooves of the track match the sprocket teeth, counteracting circumferential torque and suppressing body deviation. The outer surface is coated with a rubber anti-slip layer that enhances friction and reduces slippage on icy surfaces, while its flexible deformation protects the cable sheath and provides damping. Each locomotion module includes three sprockets—the two side sprockets serve as driving wheels and the central one as an idler. The DC-geared motor provides high torque, precise speed control, and a compact structure, driving the wheels to move the track while the idler maintains belt tension and prevents sagging. Flanges on both sides of the sprockets constrain lateral deviation, and stiffening ribs enhance torsional rigidity and vibration resistance, preventing loosening under high-frequency excitation [24].

**Figure 6:**
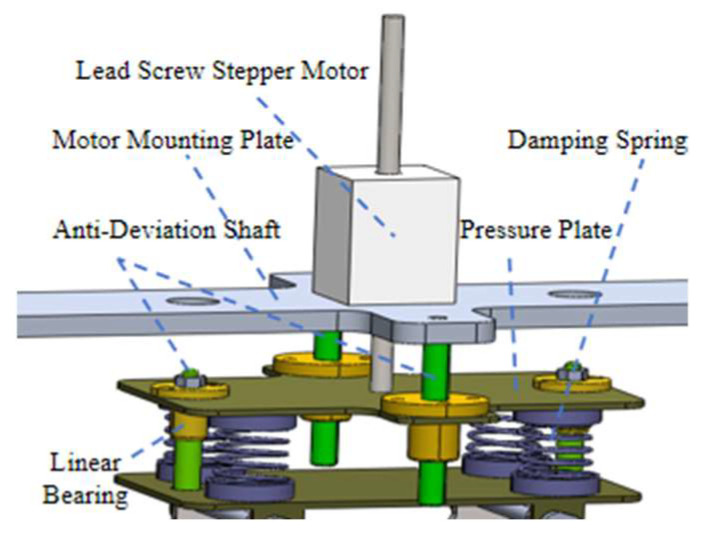
Structural diagram of the robot clamping mechanism.

As shown in Figure 6, the clamping mechanism consists of a lead screw stepper motor, a T-shaped screw, four damping springs, and linear guiding components. The stepper motor drives the lead screw to adjust the robot’s inner diameter, thereby controlling the radial position of each clamping track. When the track contacts the stay cable, the springs compress to generate a controllable clamping force. Each module is equipped with a 140 mm lead screw providing a 110 mm adjustable stroke, allowing the mechanism to accommodate stay cables with diameters from 120 mm to 230 mm. The clamping force is automatically regulated according to the detected cable diameter and can be increased under icing conditions by additional spring compression to enhance frictional adhesion. The four symmetrically arranged damping springs not only provide sufficient normal force but also absorb vibration and impact, enabling stable adaptation to the cable’s flexibility and surface irregularities. Three identical modules are positioned at 120° intervals to form a self-centering triangular configuration that maintains geometric balance during climbing. Guide rods and linear bearings ensure precise axial motion of the lead screw assembly, minimize friction, and prevent mechanical wear.

### 2.3. Analysis

Since the robot comprises three climbing modules, the opening and closing of the clamping mechanism determine its adaptability to different cable diameters. According to the design requirements, the system must accommodate stay cables with diameters ranging from 80 mm to 210 mm. To meet this requirement, each clamping unit is designed with an adjustment range of at least 130 mm, and the lead screw length is set to 160 mm.

**Figure 7:**
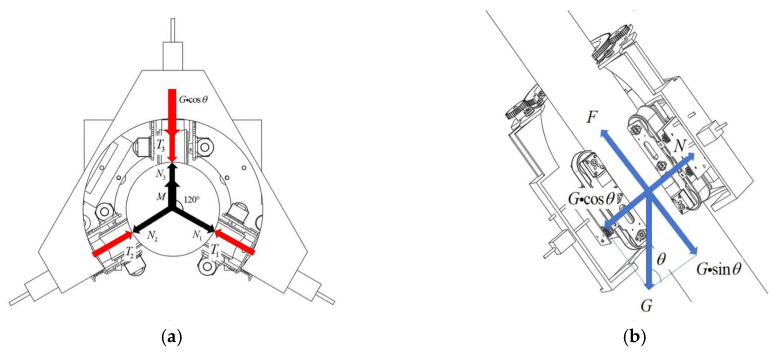
Photographs of (**a**) De-icing robot planar force diagram; (**b**) De-icing robot axial force diagram.

Figure 7a illustrates the force distribution of the stay cable de-icing robot during climbing. The black arrows indicate the reaction forces exerted by the stay cable on the robot. The clamping force generated by the robot is transmitted through three distinct reaction forces, denoted as N_1_, N_2_, and N_3_, which act on the robot structure. During climbing, the robot is subjected to gravity and aerodynamic drag. However, since the climbing speed is low, aerodynamic drag is negligible [20]. Under the configuration shown in Figure 7a, the minimum friction force F required to ensure upward climbing without slipping must satisfy:

(1) $$F=(N_{1}+N_{2}+N_{3})\;\mu \geq G\sin\theta$$

where the robot mass is m=30 kg; the gravitational force is G=300 N; θ is the inclination angle of the stay cable; $N_{n}$ is the normal force exerted by the stay cable on the robot; μ is the coefficient of friction between the stay cable and the rubber track.

As shown in Figure 7a, the reaction forces $N_{1}$, $N_{2}$, and $N_{3}$ exerted by the stay cable on the de-icing robot can be expressed as

(2) $$\left\{\begin{array}{l} N_{1}=T_{1}\\ N_{2}=T_{2}\\ N_{3}=T_{3}+G\cdot \cos\theta \end{array}\right.$$

where $F_{Tn}$ denotes the clamping force applied by the robot.

In this case:

(3) $$F_{T1}=F_{T2}=F_{T3}$$

By substituting Equations (2) and (3) into Equation (1), the minimum clamping force required to prevent the robot from slipping during upward climbing can be derived as

(4) $$F_{T1},F_{T2},F_{T3},\geq \frac{G\sin\theta }{3\mu }-\frac{G\cos\theta }{3}$$

### 2.4. Motor and Spring Selection

As shown in Figure 7b, the maximum clamping force occurs when the robot climbs vertically. Under this condition, the clamping forces in all three directions are equal. The clamping force required for a single module must satisfy:

(5) $$F_{T}\geq \frac{G}{3\mu }$$

Furthermore, the driving force provided by each motor in the drive module must satisfy:

(6) $$F_{t}>(G+3\cdot F_{T}\cdot \mu )/6=\frac{G}{3}=100\;N$$

The output torque of the motor under this condition is given by:

(7) $$F_{T}=\frac{F_{t}}{2i}\cdot \frac{D}{2\eta }$$

where D is the equivalent diameter of the driving wheel; i is the gear reduction ratio of the driving module; η is the transmission efficiency of the motor and reducer;

According to the required driving force, the minimum output torque $T_{\min}$ of each motor must satisfy: $T_{\min}>F_{t}\cdot r=3.5\;N\cdot m$.

According to the de-icing operation requirements, the robot must achieve an average speed of 10 m/min. Given that the diameter of the driving wheel is 70 mm, the required motor speed n (in revolutions per minute) must satisfy: $n\geq \frac{10}{0.07\pi }=45.5$.

To meet both the driving force and walking speed requirements of the robot, a DC geared motor was selected. The corresponding parameters are listed in Table 1.

The clamping force applied to the stay cable is generated by the lead screw stepper motor, making its selection equally important. When the surface of the stay cable is covered with ice, the motor must produce a sufficiently large clamping force to prevent the robot from slipping. According to the analysis in Section 2.3, the required clamping force reaches its maximum under vertical climbing conditions with surface icing, approximately 600 N. The specifications of the selected lead screw stepper motor are listed in Table 2.

In the clamping mechanism of the stay cable de-icing robot, the spring serves as the primary load-bearing component. During climbing, the clamping force is transmitted through the spring to the track and then applied to the stay cable. The magnitude of the spring force directly affects the climbing performance of the robot [25]. If the spring force is too large, the required driving torque of the motor will increase. If the spring force is too small, slippage or helical climbing may occur. According to the working conditions, two springs made of 65 Mn spring steel—with high strength and good machinability—were selected. The mean diameter D=22 mm and the spring index C=4 were initially determined. The wire diameter d=2.5 mm was estimated based on the spring index. The final spring dimensions were set as 2.5 mm × 22 mm × 35 mm.

Spring Stiffness:

$$k=\frac{\left(G\cdot d^{4}\right)}{\left(8\times N_{c}\cdot {D_{m}}^{3}\right)}=27.6$$

where G is the shear modulus of 65 Mn spring steel; d is the wire diameter of the spring; $D_{0}$ is the outer diameter of the spring; $D_{m}$ is the mean coil diameter; N is the total number of coils; $N_{c}$ is the number of active coils.

After the lead screw compresses the compression spring, the spring is shortened, and the generated elastic force is converted into a clamping force acting on the stay cable. Therefore, the thrust provided by the stepper motor can be calculated based on the spring deformation. The lead of the stepper motor screw is 2 mm, indicating that one full rotation of the screw results in a spring compression of 2 mm upon track contact with the stay cable. The corresponding clamping force T=k⋅2 mm leads to an increase in the friction force between the robot and the stay cable, which can be calculated as follows:

(8) ΔF=6k⋅μ

## 3. Climbing Mechanism Speed Control

The core objective of climbing motion control for stay cable de-icing robots is to maintain coordinated stability between speed and clamping force. Since the robot ascends unidirectionally along a fixed cable without requiring steering capability, the control logic primarily emphasizes precise speed tracking and adaptive adjustment of clamping force. In winter, the surface icing conditions of stay cables become highly variable, and when the robot enters an ice-covered section, the friction coefficient between the robot and the cable may decrease sharply, increasing the risk of instability or even slippage. Therefore, real-time regulation of the clamping force is essential to ensure stable and reliable de-icing performance.

### 3.1. Speed-Clamp Relation

The climbing function of the robot is powered by a DC geared motor. Variations in the friction between the robot and the stay cable directly affect the load applied to the motor. As the load increases, the motor output torque rises correspondingly, leading to a decrease in rotational speed. Conversely, a lower load results in a higher motor speed. If the clamping mechanism fails to generate sufficient friction, the robot may lose traction or even slip downward. Therefore, regulating the friction between the robot and the stay cable is essentially equivalent to controlling the load applied to the DC geared motor. When the motor load reaches a predetermined value, it signifies that the frictional force has achieved the required level.

The relationship between the output torque and the rotational speed of the DC- geared motor can be expressed as

$$T=\frac{9.55P}{n}$$

where  T denotes the output torque;  P represents the output power; and n is the motor speed.

The climbing speed of the robot along the stay cable is given by:

(9) V=n⋅2R⋅π

where n denotes the rotational speed of the DC geared motor, which is affected by the load and thus influences the climbing velocity of the robot.

The relationship between the motor output torque and the tangential load can be expressed as

(10) $$T=F_{t}\cdot R$$

where R is the radius of the driving wheel, and $F_{t}$ is the tangential load acting on the wheel that must be overcome by the motor.

(11) $$F_{t}=(N_{1}+N_{2}+N_{3})\cdot \mu -G\cdot \sin\theta$$

By combining Equations (1) and (2), the traction force $F_{t}$ can be expressed as:

(12) $$F_{t}=(3F_{T}+G\cdot \cos\theta )\cdot \mu -G\cdot \sin\theta$$

The clamping force $F_{T}$ is related to the number of screw rotations $\Delta_{r}$ as follows:

(13) $$F_{T}=2\cdot \Delta_{r}\cdot k$$

The relationship between the motor speed V and the number of screw rotations is given by:

(14) $$V=\frac{19.1\cdot \pi \cdot P}{(6\cdot \Delta_{r}\cdot k+G\cdot \cos\theta )\cdot \mu -G\sin\theta }$$

As indicated by the equation, when the surface of the stay cable becomes smoother (i.e., when the friction coefficient decreases), a greater number of screw rotations is required to increase the clamping force, thereby maintaining the robot’s climbing stability and regulating its speed.

### 3.2. Fuzzy Pid-Based Clamping Control

**Figure 8:**
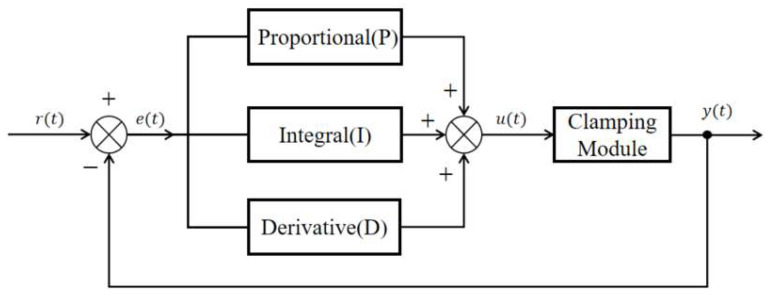
Block diagram of the conventional PID control principle.

The PID controller regulates the controlled system in real time by comparing the error between the input and the output and by linearly combining the proportional, integral, and derivative components. It is characterized by a simple structure, strong robustness, and high adaptability to parameter variations and external disturbances [26]. In the stay cable de-icing robot, the PID control system takes the geared motor speed as the control target and adjusts the clamping force of the clamping module in real time to balance the load and maintain a stable motor speed. The working principle and signal flow of the control loop are illustrated in Figure 8.

In Figure 8, the error signal e(t) is defined as the difference between the reference input r(t) and the actual output y(t) of the controller:

(15) e(t)=r(t)−y(t)

The PID control law is expressed as

(16) $$u(t)=k_{p}e(t)+k_{i}\int_{0}^{t}e(t)dt+k_{d}\frac{de(t)}{\mathrm{dt}}$$

where u(t) is the control signal for the clamping mechanism; $k_{p}$, $k_{i}$, and $k_{d}$ are the proportional, integral, and derivative gains, respectively.

In a conventional PID control system, these three parameters work together to achieve trajectory tracking of the robot. However, since only a fixed set of parameters is used, the controller cannot account for the robot’s nonlinear characteristics or external disturbances [27]. As a result, both the motion accuracy and stability of the robot can be significantly affected.

**Figure 9:**
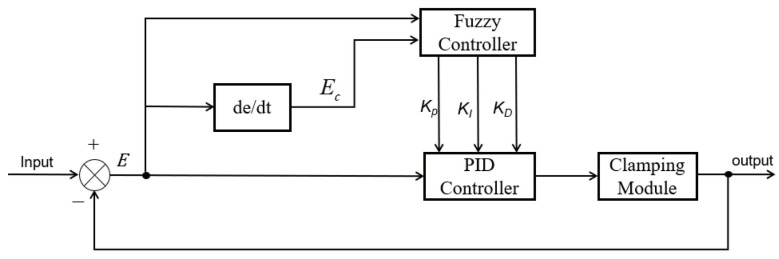
Block diagram of the fuzzy PID control principle.

To overcome the limitations of conventional PID control, this study proposes a fuzzy adaptive PID control algorithm for regulating the clamping force of the stay cable de-icing robot. The algorithm incorporates a fuzzy control module into the traditional PID controller, enabling real-time adjustment of the three PID parameters based on the system’s dynamic state. The overall structure of the fuzzy PID controller is illustrated in Figure 9.

The fuzzy PID control system regulates the coordination between speed and clamping force of the stay cable de-icing robot as follows: First, the real-time speed of the geared motor obtained by the system is compared with the preset reference speed to calculate the error e and its rate of change $e_{c}$. These two quantities are then used as input variables for the fuzzy controller, where they undergo fuzzification. After fuzzification, the inputs are processed according to predefined fuzzy rules and membership functions to generate fuzzy output sets. Through defuzzification, precise output values are obtained. The fuzzy controller outputs the variation increments of $\Delta k_{p}$, $\Delta k_{i}$, and $\Delta k_{d}$, which are used to adaptively tune the parameters of the PID controller in real time. Finally, the adjusted PID controller outputs the control signal to the clamping module.

### 3.3. Fuzzification of Input and Output Control Variables

**Figure 10:**
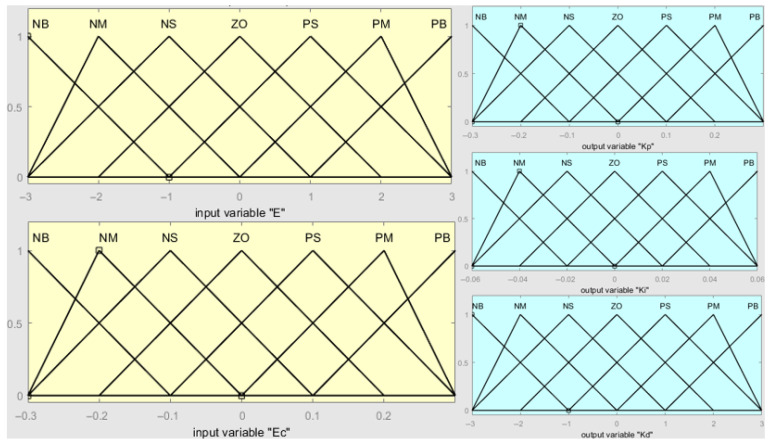
The membership function diagrams.

In the fuzzy control system designed in this study, different icing and snow-covered conditions were applied to the cable surface under the same inclination angle of the stay cable. The velocity error was obtained by comparing the robot’s actual climbing speed with the preset reference speed. The fuzzy PID controller takes the velocity error (e) and its rate of change ($e_{c}$) as input variables, while $k_{p}$, $k_{i}$, and $k_{d}$ serve as output variables. These five variables are converted into seven linguistic variables: NB (Negative Big), NM (Negative Medium), NS (Negative Small), ZO (Zero), PS (Positive Small), PM (Positive Medium), and PB (Positive Big). This process represents the fuzzification of both input and output variables. According to the design requirements, the universes of discourse are defined as [−3, 3] for e and [−0.3, 0.3] for $e_{c}$, while those for the fuzzy controller output variables $k_{p}$, $k_{i}$ and $k_{d}$ are [−0.3, 0.3], [−0.06, 0.06], and [−3, 3], respectively. The fuzzy control system developed in this study adopts a triangular membership function, which offers high flexibility and ease of implementation. By inputting the domain range of each parameter, the corresponding membership functions of each input and output variable are obtained, as shown in Figure 10.

The membership functions of the input variables (e and $e_{c}$) and the output variables ($\Delta k_{p}$, $\Delta k_{i}$, and $\Delta k_{d}$) are illustrated in Figure 10.

The membership function is expressed as shown in Equation (17).

(17) $$\sigma \left\{\begin{array}{c} 0\\ \left(x-a)/(b-a\right)\\ \left(c-x)/(c-b\right) \end{array}\right.\begin{array}{l} x<a,x>c\\ a\leq x<b\\ b\leq x\leq c \end{array}$$

Based on the principles of fuzzy control, the fuzzy PID control rules were summarized from multiple experiments and simulation analyses, and the specific tuning principles are as follows:

(1) When the rate of error change $e_{c}$ is large and has the same sign as the error e, it indicates that the actual speed is significantly higher than the desired speed, suggesting an excessive system output. In this case, $k_{p}$ and $k_{i}$ should be reduced, while $k_{d}$ should be increased.
(2) When $e_{c}$ is small, the actual speed is close to or slightly above the desired value. To reduce overshoot, $k_{i}$ should be increased, and $k_{p}$ can be slightly adjusted to suppress internal and external disturbances.
(3) When $e_{c}$ is moderate, the output control is considered appropriate, and the clamping mechanism should maintain the current state.
(4) When $e_{c}$ is large but opposite in direction to e, it implies that the clamping force is insufficient. In this case, $k_{i}$ and $k_{d}$ should be increased to restore stability and climbing speed.

Based on the above rules, the fuzzy control rules for $\Delta k_{p}$, $\Delta k_{i}$, and $\Delta k_{d}$ are defined as shown in Table 3, Table 4 and Table 5.

By using e and $e_{c}$ as input variables of the fuzzy controller, the fuzzy relationships between $\Delta k_{p}$, $\Delta k_{i}$, and $\Delta k_{d}$ and the inputs are established based on the fuzzy control rules, as shown in Equation (18).

(18) $$\left\{\begin{array}{l} \Delta k_{p}=\left[\sum_{i=1}^{n}\sigma (K_{pi})\cdot K_{pi}\right]/\sum_{i=1}^{n}\sigma (K_{pi})\\ \Delta k_{i}=\left[\sum_{i=1}^{n}\sigma (K_{ii})\cdot K_{ii}\right]/\sum_{i=1}^{n}\sigma (K_{ii})\\ \Delta k_{d}=\left[\sum_{i=1}^{n}\sigma (K_{di})\cdot K_{di}\right]/\sum_{i=1}^{n}\sigma (K_{di}) \end{array}\right.$$

During system operation, the input variables e and $e_{c}$ are monitored in real time, and the PID control parameters $k_{p}$, $k_{i}$, and $k_{d}$ are adjusted based on fuzzy logic principles to meet the clamping force requirements under different values of e and $e_{c}$, as shown in Equation (19).

(19) $$\left\{\begin{array}{l} k_{p}=k_{p0}+\Delta k_{p}\\ k_{i}=k_{i0}+\Delta k_{i}\\ k_{d}=k_{d0}+\Delta k_{d} \end{array}\right.$$

where $k_{p0}$, $k_{i0}$, and $k_{d0}$ are the initial values of the PID parameters, while $\Delta k_{p}$, $\Delta k_{i}$, and $\Delta k_{d}$ are the outputs of the fuzzy controller.

(20) $$u(t)=A\cdot e_{w}(t)-B\cdot e_{w}(t-1)+Ce_{w}(t-2)$$

where $\left\{\begin{array}{l} A=K_{p}+K_{i}+K_{d}\\ B=K_{p}+2K_{d}\\ C=K_{d} \end{array}\right.$

**Figure 11:**
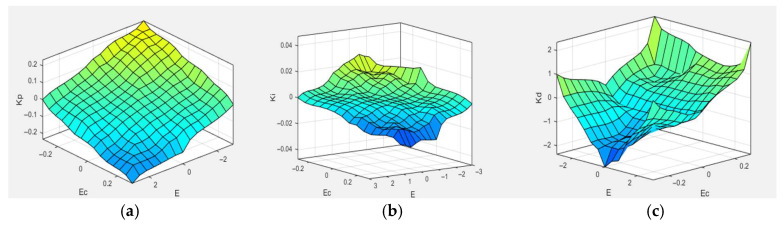
(**a**) Universe of discourse for the proportional gain (K_p_); (**b**) Universe of discourse for the integral gain (K_i_); (**c**) Universe of discourse for the derivative gain (K_d_).

To make the fuzzy controller more suitable for the clamping system of the stay cable de-icing robot, the PID parameters $k_{p}$, $k_{i}$, and $k_{d}$ are adjusted in real time during the control process. Based on the fuzzy PID control rules and membership functions of each fuzzy subset, the output surfaces of the proportional, integral, and derivative parameters over the defined domain are obtained using MATLAB, as shown in Figure 10. where (a), (b), and (c) represent the output curves of the proportional, integral, and derivative coefficients over the defined domain. It can be observed that as both e and $e_{c}$ increase, $k_{p}$ and $k_{i}$ gradually decrease, while $k_{d}$ first decreases and then increases. As shown in Figure 11, the control surfaces exhibit regular and nonlinear variations, indicating that the fuzzy control rules proposed in this study are well-suited for the clamping control requirements of the stay cable de-icing robot.

## 4. Simulation and Experimental

### 4.1. Simulation

**Figure 12:**
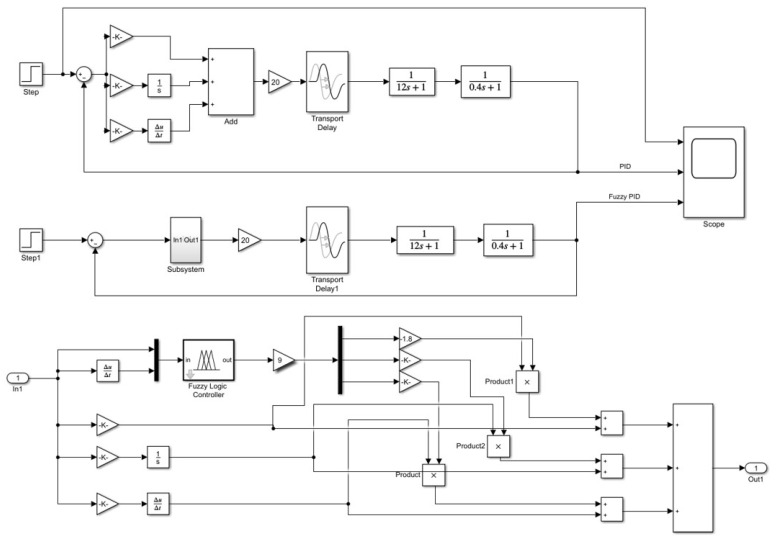
Simulation models of conventional PID and fuzzy PID controllers.

To evaluate the performance and robustness of the fuzzy PID controller applied to the climbing mechanism of the stay cable de-icing robot, two control strategies were modeled in MATLAB/Simulink: a conventional PID controller and a fuzzy PID controller. A step signal with an amplitude of 0.01 m was introduced to represent the target climbing speed. The corresponding system responses under each control scheme were subsequently analyzed, as presented in Figure 12. 

**Figure 13:**
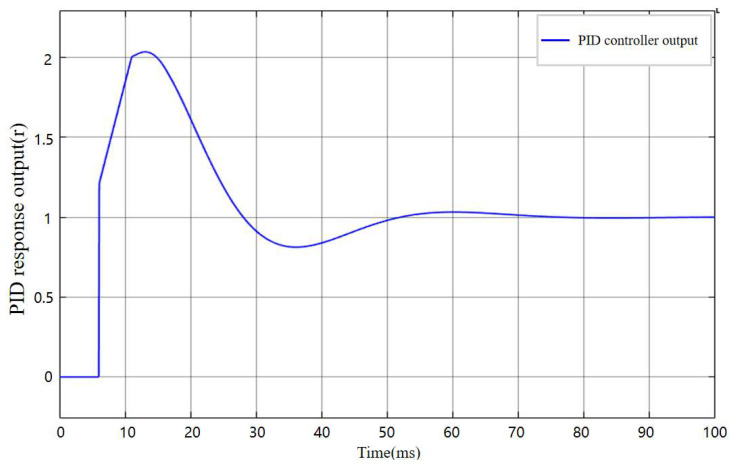
Output response curve of the PID controller under the original parameters.

The setting of the three parameters $k_{p}$, $k_{i}$, and $k_{d}$ is the core issue in control system design, as they directly affect the system’s stability, response speed, and anti-interference capability. Before determining the final values, parameter tuning needs to be performed within the PID controller. The sampling time was set to 0.1 s, and the initial control coefficients were set as $k_{p}\;=0.6$, $k_{i}\;=0.8$, and $k_{d}\;=0.03$. The simulated output response curve of the PID control system is shown in Figure 13.

**Figure 14:**
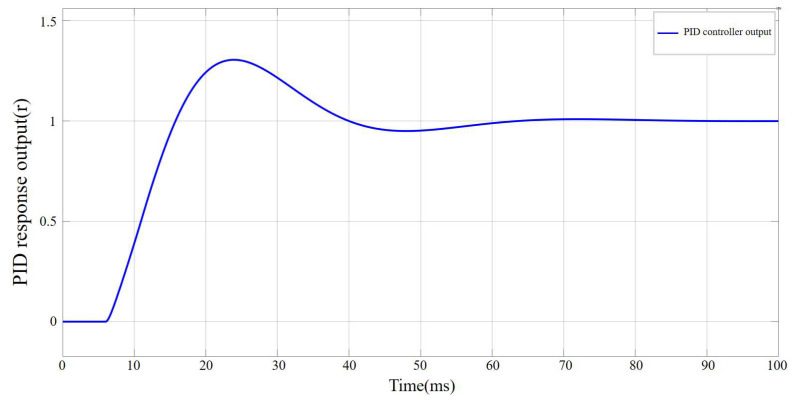
Output response curve of the PID controller after parameter tuning.

As shown in Figure 13, before parameter tuning, the PID controller response output exhibits an excessive abrupt change in the initial stage, exceeding the control target by approximately 100%, and during the steady-state convergence stage, the maximum oscillation amplitude exceeds the target by about 15%. This behavior is likely caused by an excessively large $k_{p}$, which results in an overly rapid response. Therefore, subsequent tuning primarily focused on adjusting $k_{p}$ and the final control parameters were determined as $k_{p}\;=0.34$, $k_{i}\;=0.7$, and $k_{d}\;=0.03$. The output response curve of the PID controller after parameter tuning is shown in Figure 14.

**Figure 15:**
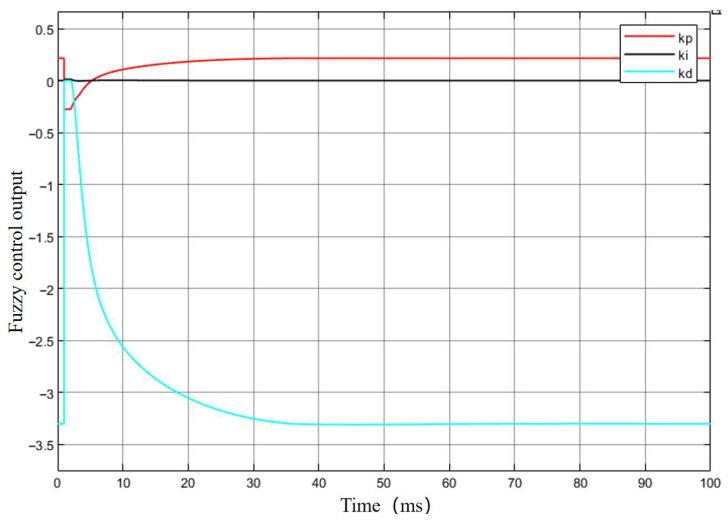
Output response curve of the fuzzy controller.

After completing the parameter adjustment, the output of the fuzzy control component in the fuzzy PID controller was tested, and the corresponding output response curve is shown in Figure 15.

As shown in Figure 15, among the three output variables of the fuzzy controller, $k_{p}$ and $k_{d}$ exhibit relatively large fluctuations at the beginning, indicating that they play a dominant role in the adjustment process, which is consistent with the fuzzy control rules. The value of $k_{p}$ becomes stable after approximately 0.2 s, and $k_{d}$ stabilizes after about 0.3 s, suggesting that the overall control system completes its adjustment signal output at around 0.3 s.

**Figure 16:**
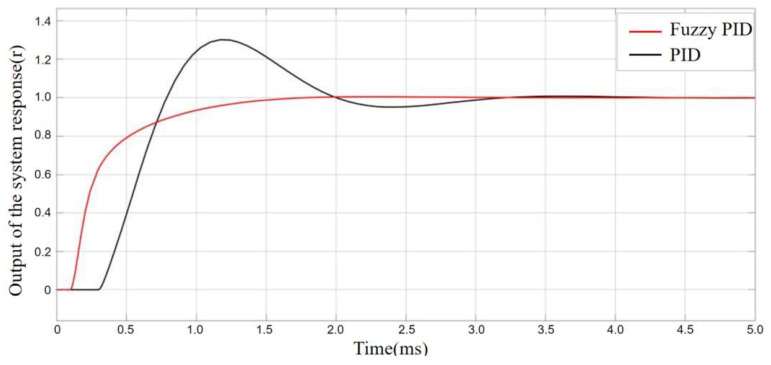
Performance comparison of PID and Fuzzy-PID.

Based on the simulation model constructed in Figure 12, the output response curves of the de-icing robot under the conventional PID and fuzzy PID controllers without external disturbance are shown in Figure 16. The black curve represents the control performance of the conventional PID controller, while the red curve represents that of the fuzzy PID controller. As can be seen from the simulation results, the conventional PID controller exhibits an overshoot exceeding 20%, with a settling time greater than 0.6 s, and the curve shows noticeable oscillations. In contrast, the fuzzy PID controller achieves a response curve that follows the reference trajectory more closely at the inflection points, effectively avoiding the overshoot problem observed in the conventional PID controller. Moreover, the fuzzy PID controller significantly improves the system’s response speed, reducing the response time by approximately 50%.

**Figure 17:**
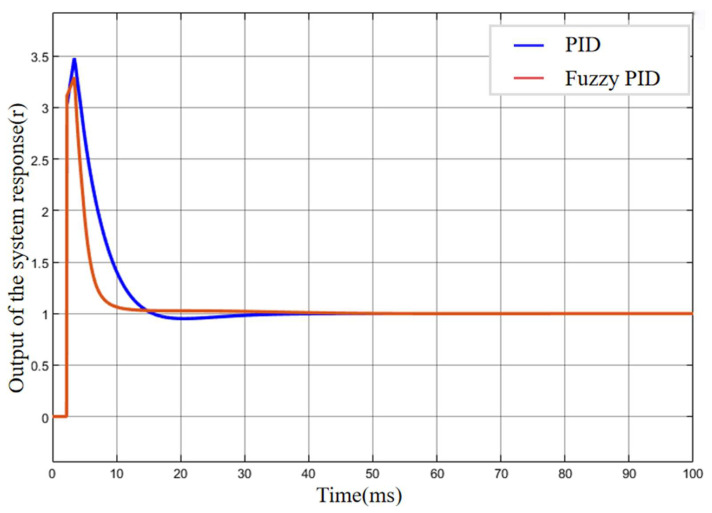
Output response curves of the two controllers after introducing the delay block.

To further verify the superiority of the fuzzy PID control compared with the traditional PID control, a delay module was introduced into the Simulink control model. The delay period was set to 1, and the initial buffer size was configured to 500 data points. As shown in Figure 17, after introducing the delay module, the system exhibited a sharp transient response in the initial stage, with a peak value exceeding the target by approximately 350%. However, within 0.05 s, the outputs of both controllers began to converge rapidly toward the target. The fuzzy PID controller reached the desired value within approximately 0.1 s without overshoot, while the conventional PID controller required around 0.3 s and exhibited a noticeable overshoot. These results demonstrate that the fuzzy PID controller maintains strong robustness in the presence of system delay and is thus more suitable for practical scenarios involving delay-induced parameter fluctuations.

### 4.2. Experimental

To evaluate the dynamic response capability of the fuzzy PID control system under sudden changes in the friction coefficient of stay cable surfaces, a series of climbing experiments under varied friction conditions were conducted. These experiments were designed to determine whether the control system could adaptively regulate the clamping force in real time based on the operating state of the DC geared motor, thereby ensuring stable control of the climbing speed. Tests were carried out on stay cables inclined at 30°, 45°, and 60°, under two typical scenarios of abrupt friction transition: from ice-free to ice-covered and from ice-covered to ice-free. These setups were designed to simulate disturbances encountered under varying icing conditions. During climbing, the upper driving module bore the main gravitational component, and its load fluctuations had a significant impact on system stability. Therefore, it was selected as the primary control target. To improve control efficiency, the robot was installed in a downward-facing orientation, with the upper DC geared motor designated as the master motor. Its rotational speed served as the global reference, enabling coordinated control adjustments based on the node experiencing the highest load and preventing motor overshoot caused by insufficient load.

**Figure 18:**
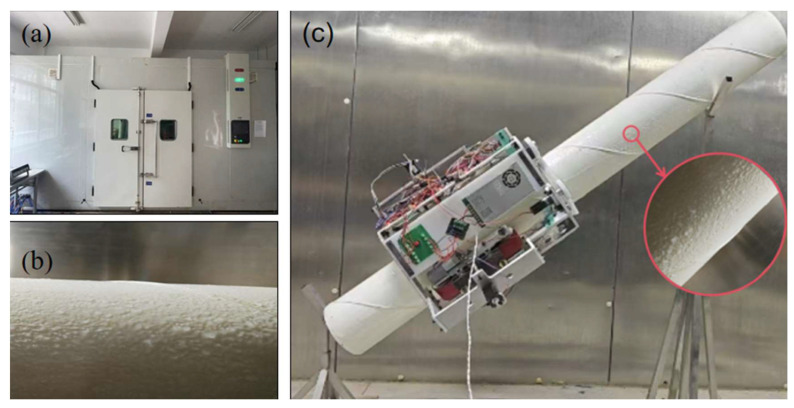
Photographs of (**a**) stepped constant-temperature and constant-humidity chamber; (**b**) ice layer on the surface of the stay cable; (**c**) prototype test of the stay cable de-icing robot.

For experimental validation, the testing conditions were established according to the typical icing environment of the Erqi Yangtze River Bridge in Wuhan during winter. The indoor temperature, cable inclination, and cooling procedures were configured to replicate actual field conditions. The experimental process included the following steps: first, a PE-sheathed stay cable was vertically positioned inside a temperature- and humidity-controlled chamber until the ambient temperature reached −5 °C. Then, water cooled to 0–1 °C was sprayed from multiple angles onto the lower half of the sheath to form an approximately 3 mm-thick ice layer. After freezing was complete, the sheath was mounted on a support frame with the iced section facing upward. Next, the robot was clamped to the lower, ice-free section using the clamping mechanism (as shown in Figure 18), and the climbing speed was set to 10 m/min. The rotational speed of the DC geared motor was recorded before and after entering the ice-covered section; the nominal motor speed was 45.5 r/min. The test was then repeated with the ice-covered section positioned at the bottom to simulate the ice-covered-to-ice-free transition. Each test was conducted at different cable inclinations to obtain comparative data on the system response.

**Figure 19:**
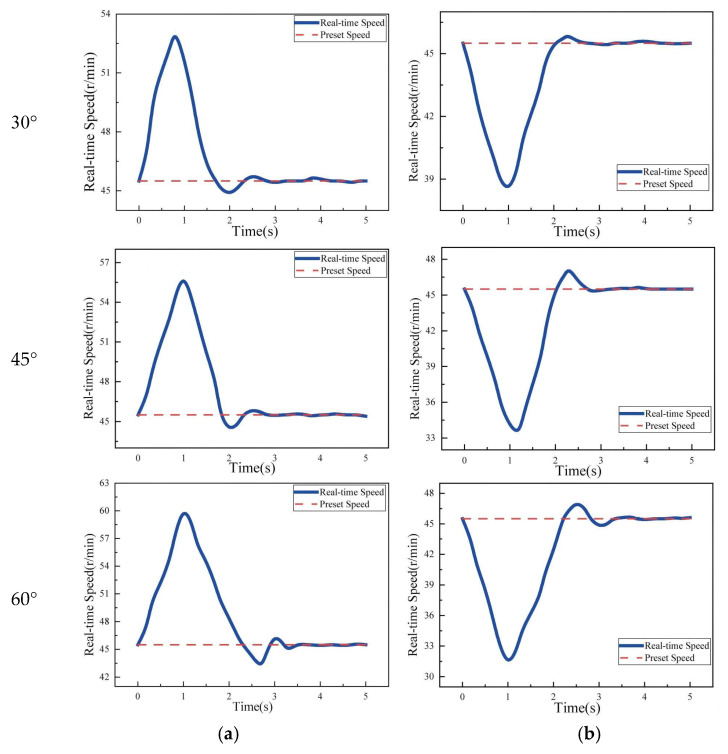
(**a**) Real-time speed at different angles from ice-free segment to iced segment, (**b**) real-time speed at different angles from iced segment to ice-free segment.

The time-dependent rotational speed curves of the DC geared motor under different slope conditions are shown in Figure 19.

Experimental data show that when a sudden change in the friction coefficient occurs on the stay cable surface, the rotational speed of the DC geared motor exhibits a three-stage dynamic response:

In the first stage—the sudden change phase—insufficient clamping force causes a loss of friction torque balance, leading to a sharp increase in motor speed and the appearance of a peak value. In the second stage—the rapid adjustment phase—the fuzzy PID controller drives the lead screw stepper motor to compress the shock-absorbing spring, thereby increasing the friction force between the robot and the cable. This increases the motor load and causes the motor speed to converge toward the preset target. In the third stage—the steady-state convergence phase—a slight overshoot occurs after the motor speed reaches the reference value. After slight oscillations, the speed stabilizes within the preset range, with a steady-state error of less than ±1.5%, confirming the dynamic convergence capability of the control system.

**Figure 20:**
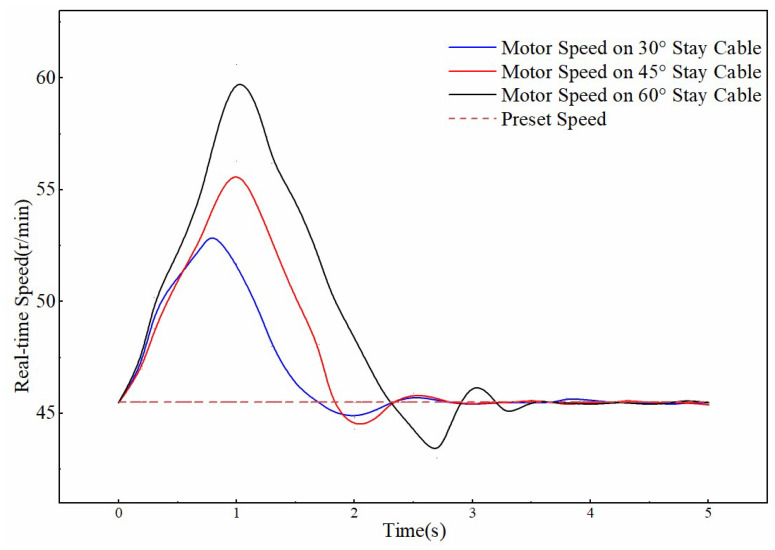
Speed variation from ice-free to icy sections under different angles.

Taking the transition from an ice-free surface to an ice-covered surface as an example, the effect of cable inclination on climbing control performance is summarized in Figure 20 and Table 6. The data indicate that a larger inclination angle leads to a greater gravitational component along the cable, intensifying the torque imbalance during the friction coefficient mutation. Consequently, the speed spike increases (the peak speed at a 60° incline is 15% higher than at 30°). At steeper inclines, the system must compensate for larger gravitational torque and friction loss, resulting in longer motor regulation time (approximately 4 s at 60°, which is a 60% increase compared to 30°). In high-inclination scenarios, the increased control difficulty of the clamping module leads to higher parameter sensitivity, and the overshoot of motor speed also increases with the inclination angle (the overshoot at 60° is approximately 110% higher than that at 30°).

Climbing experiments on the stay cable de-icing robot demonstrate that the proposed mechanical structure is stable, reliable, and exhibits smoothly operating, achieving the expected climbing speed. When the surface conditions of the stay cable change or complex scenarios such as ice and snow accumulation occur, the robot’s intelligent control system can rapidly detect and respond to environmental variations, adaptively adjusting the climbing strategy with high precision and responsiveness.

The robot maintained favorable dynamic performance and stability throughout the tests under various cable inclinations and environmental conditions, confirming its practical applicability and robustness. Thees results provide critical technical support for developing intelligent, automated, high-altitude cable maintenance equipment. Furthermore, they establish a solid technical foundation for the safe operation and maintenance of large-scale infrastructure such as cable-stayed bridges, offering significant engineering application value and promising prospects for future implementation.

## 5. Conclusions

To address the challenges of poor climbing performance and slippage encountered by cable-stayed bridge de-icing robots due to smooth and unevenly iced cable surfaces, this study proposes a three-point encircling, track-based drive mechanism and a real-time self-adjusting clamping mechanism. These structural features enhance the robot’s adhesion to the cable surface. Furthermore, a fuzzy PID control algorithm is implemented to provide adaptive regulation of the clamping force, enabling the robot to maintain a stable and uniform climbing speed under varying surface conditions.

To validate the feasibility of the proposed climbing mechanism and the effectiveness of the fuzzy PID control strategy, a MATLAB/Simulink-based simulation model was developed, and a prototype robot was fabricated for physical climbing tests. Both simulation and experimental results demonstrate that the proposed system can automatically adjust the clamping force in real time, maintaining climbing stability across cables with different surface smoothness and icing conditions. Compared with a conventional PID controller, the fuzzy PID reduced response time by approximately 50%, keeping the speed error within ±1.5%. Additionally, the fuzzy PID controller exhibits nonlinear adaptive capability, allowing real-time tuning of control parameters in response to environmental uncertainties and external disturbances. Compared to traditional PID controllers, the fuzzy PID approach maintains control quality under system parameter variations and fluctuating loads.

Therefore, the proposed design and control strategy provides a technical solution and basis for the practical deployment of de-icing robots on stay cables under complex real-world conditions.

## 6. Discussion

The modeling in Equations (1)–(7) simplified dynamic friction and neglected cable curvature and vibration effects, which may lead to unmodeled instabilities under real bridge conditions. Future work will refine the model by incorporating curvature-dependent contact and vibration effects. In addition, the controller’s robustness under noise, motor delay, and load variations has not yet been evaluated. Future studies will introduce disturbance and uncertainty models to evaluate the controller’s adaptive stability and robustness. The experimental validation was limited to indoor static cable tests and did not account for wind or temperature fluctuations. As the study is currently limited to the laboratory stage, future work will involve outdoor and dynamic experiments to evaluate the system’s performance under realistic conditions.

## Figures and Tables

**Table 1:** Specifications of the 5882-50ZY DC geared motor(Shenzhen XYT Motor Co., Ltd., Shenzhen, China).

Reduction Ratio	Rated Voltage (V)	Rated Torque (kg.cm)	Power (W)	No-load Speed (RPM)	Rated Speed (RPM)
48	24	40	108	120	86

**Table 2:** Specifications of the 57 × 76 lead screw stepper motor.

Holding Torque (mN·m)	Max. Horizontal Load (kg)	Max. Vertical Load (kg)	Screw Size (mm)	Lead (mm)	Full-load Speed (450 rpm/min)
1900	54	45	0.63	2	15.0 mm/s

**Table 3:** Fuzzy control rules for $k_{p}$.

		e						
		NB	NM	NS	ZO	PS	PM	PB
e_c_	NB	PB	PB	PM	PM	PS	ZO	ZO
	NM	PB	PB	PM	PS	PS	ZO	NS
	NS	PM	PM	PM	PS	ZO	NS	NS
	ZO	PM	PM	PS	ZO	NS	NM	NM
	PS	PS	PS	ZO	NS	NS	NM	NM
	PM	PS	ZO	NS	NM	NM	NM	NB
	PB	ZO	ZO	NM	NM	NM	NB	NB

**Table 4:** Fuzzy control rules for $k_{i}$.

		e						
		NB	NM	NS	ZO	PS	PM	PB
e_c_	NB	NB	NB	NM	NM	NS	ZO	ZO
	NM	NB	NB	NM	NS	NS	ZO	ZO
	NS	NB	NM	NS	NS	ZO	PS	PS
	ZO	NM	NM	NS	ZO	PS	PM	PM
	PS	NM	NS	ZO	PS	PS	PM	PB
	PM	ZO	ZO	PS	PS	PM	PB	PB
	PB	ZO	ZO	PS	PM	PM	PB	PB

**Table 5:** Fuzzy control rules for $k_{d}$.

		e						
		NB	NM	NS	ZO	PS	PM	PB
e_c_	NB	PS	NS	NB	NB	NB	NM	PS
	NM	PS	NS	NB	NS	NS	ZO	ZO
	NS	ZO	NS	NM	NS	ZO	PS	PS
	ZO	ZO	NS	NS	ZO	PS	PM	PM
	PS	ZO	ZO	ZO	PS	PS	PM	PB
	PM	PB	NS	PS	PS	PM	PB	PB
	PB	PB	PM	PM	PM	PM	PB	PB

**Table 6:** Effect of inclination angle on climbing control performance.

Inclination Angle (°)	Peak Rotational Speed (r/min)	Speed Regulation Time (s)	Rise Time (s)	Steady-State Error (%)	Overshoot (r/min)	Climbing Duration (s)
30°	53.2	2.5	0.748	<±1.5	0.7	13.6
45°	56.3	3.0	0.801	<±1.5	1.2	15.8
60°	60.6	3.6	0.794	<±1.5	2.3	17.2

## Data Availability

The datasets and material used during this study are available from the corresponding authors upon reasonable request.
